# Poor Sleep Quality Is Associated with Dawn Phenomenon and Impaired Circadian Clock Gene Expression in Subjects with Type 2 Diabetes Mellitus

**DOI:** 10.1155/2017/4578973

**Published:** 2017-03-02

**Authors:** Yuxin Huang, Haidong Wang, Yuan Li, Xiaoming Tao, Jiao Sun

**Affiliations:** ^1^Department of Endocrinology, Huadong Hospital Affiliated to Fudan University, 221 Yananxi Road, Shanghai 200040, China; ^2^Shanghai Key Laboratory of Clinical Geriatric Medicine, Shanghai, China

## Abstract

*Aims*. We investigated whether poor sleep quality is associated with both dawn phenomenon and impaired circadian clock gene expression in subjects with diabetes. *Methods*. 81 subjects with diabetes on continuous glucose monitoring were divided into two groups according to the Pittsburgh Sleep Quality Index. The magnitude of dawn phenomenon was quantified by its increment from nocturnal nadir to prebreakfast. Peripheral leucocytes were sampled from 81 subjects with diabetes and 28 normal controls at 09:00. Transcript levels of circadian clock genes (*BMAL1*, *PER1*, *PER2*, and *PER3*) were determined by real-time quantitative polymerase chain reaction. *Results.* The levels of HbA1c and fasting glucose and the magnitude of dawn phenomenon were significantly higher in the diabetes group with poor sleep quality than that with good sleep quality. Peripheral leucocytes from subjects with poor sleep quality expressed significantly lower transcript levels of *BMAL1* and *PER1* compared with those with good sleep quality. Poor sleep quality was significantly correlated with magnitude of dawn phenomenon. Multiple linear regression showed that sleep quality and *PER1* were significantly independently correlated with dawn phenomenon. *Conclusions*. Dawn phenomenon is associated with sleep quality. Furthermore, mRNA expression of circadian clock genes is dampened in peripheral leucocytes of subjects with poor sleep quality.

## 1. Introduction

The term “dawn phenomenon” was first introduced by Schmidt et al. in 1981 and was used to describe fasting hyperglycemia or a spontaneous rise in insulin requirements during the early morning, occurring in the absence of nocturnal hypoglycemia [1]. Dawn phenomenon mostly affects children and youth with type 1 diabetes, although it is not rare in subjects with type 2 diabetes (as high as 40%, reported by Monnier et al.) [2]. By using a continuous glucose monitoring system, dawn phenomenon could be determined by the difference between nocturnal nadir and prebreakfast glucose levels [2]. Monnier et al. published that the approximate impact of the dawn phenomenon on HbA1c level was 0.4% and the impact for averaged 24 h mean glucose concentrations was 12.4 mg/dL [3]. Furthermore, fasting glucose levels are one of the most important targets in the management of type 2 diabetes, and these can be affected by the dawn phenomenon. Thus, we should not ignore this problem.

The mechanism of dawn phenomenon still remains unclear. It is generally believed that dawn phenomenon is a result of pancreatic beta-cell dysfunction, which increases endogenous glucose production, persistent insulin resistance, and hepatic glucose output. Some endogenous hormones, especially growth hormone, cortisol, and glucagon, also take part in the pathophysiological mechanism of dawn phenomenon [4, 5]. In addition, age was reported to have an independent effect on the dawn phenomenon [2]. It is well known that poor sleep quality is common in elderly subjects. Sleep disorder is associated with insulin resistance and impaired glucose tolerance in multiple metabolic pathways in healthy subjects or in subjects with diabetes [6]. Furthermore, obstructive sleep apnea-hypopnea syndrome (OSAHS) is also associated with insulin resistance, and it is common in subjects with diabetes. Thus, we believe poor sleep quality is one of the core mechanisms in dawn phenomenon.

On the other hand, mechanisms that impair the circadian clock genes, such as circadian locomotor output cycles kaput (*CLOCK*), brain and muscle Arnt-like protein 1 (*BMAL1*), and period genes (*PER1*, *PER2*, and *PER3*), contribute to defective beta-cell function and development of type 2 diabetes [7–12]. In mammals, the circadian system exists in almost all tissues, including hypothalamic suprachiasmatic nucleus (SCN, regarded as the central clock), liver, pancreas, kidney, and leucocytes [13]. Circadian clock has a vital impact on the regulation of physiological and biochemical processes, cellular and energy metabolism, glucose and lipid homeostasis, and feeding behavior. Disruption of these clock genes affects locomotor activity, feeding behavior, metabolism, and glucose homeostasis [14]. Therefore, we speculate that disruption of circadian clock genes may also indirectly increase glucose levels during the early morning.

Publications have shown that circadian clock genes are associated with sleep outcomes in human research [15, 16]. Therefore, it is interesting to find an interaction between dawn phenomenon, sleep quality, and circadian clock gene expression in subjects with diabetes. To the best of our knowledge, there is a lack of robust evidence to support this relationship. Thus, we felt it was imperative to assess this issue. In the present study, we investigated the following: (a) whether sleep quality is associated with dawn phenomenon and (b) whether dawn phenomenon is correlated with impaired circadian clock gene expression in subjects with type 2 diabetes. These results may help us to recognize a new pathophysiological mechanism of dawn phenomenon and find a new target for management and treatment of type 2 diabetes.

## 2. Materials and Methods

All participants were recruited from Department of Endocrinology, Huadong Hospital Affiliated to Fudan University, Shanghai, People's Republic of China, from March 2014 to July 2015. There were, in total, 81 subjects with type 2 diabetes with poor sleep quality or with good sleep quality and 28 normal controls with good sleep quality enrolled in the study according to the protocol. The study protocol was approved by the Ethics Committee of Huadong Hospital. All procedures performed in studies involving human participants were in accordance with the ethical standards of the institutional research committee and with the 1964 Helsinki declaration. Informed consent was obtained from all individual participants included in the study.

### 2.1. Inclusion Criteria

The inclusion criteria were comprised of the following.

(1) All participants with diabetes were diagnosed with type 2 diabetes according to the 2014 American Diabetes Association criteria [17]. (2) All subjects with diabetes received stable treatment with dietary specifications alone or with oral hypoglycemic agents (OHAs, including metformin, *α*-glucosidase inhibitors, sulfonylureas, glinides, and dipeptidyl peptidase-4 inhibitors or in a combination of 2 OHAs) for at least 3 months.

Normal controls were determined to be healthy participants without diabetes, impaired glucose tolerance, poor sleep quality, or a history of OSAHS.

### 2.2. Exclusion Criteria

The exclusion criteria included the following.

(1) Subjects received insulin therapy in 3 months prior to the study; (2) they had an HbA1c level of more than 75 mmol/mol (9.0%); (3) they were currently afflicted with diabetic ketoacidosis or hyperosmolar coma; (4) they were currently afflicted with cardiovascular disease or other serious diseases; (5) they were currently afflicted with hypoglycemia or suspected hypoglycemia; (6) they had a creatinine clearance rate of <45 mL/min; (7) they had impaired liver function (liver enzymes more than twice the upper limit of normal); (8) they were currently receiving any hypnagogue; (9) they should be in poor compliance with diabetes treatment; or (10) they had a history of OSAHS.

### 2.3. Clinical Investigations and Laboratory Determinations

All participants were instructed to maintain their recommended dietary, medication, and exercise programs. Following a 10 h overnight fast, anthropometric, blood pressure, serum, and plasma samples were collected. Biochemical measurements of plasma glucose, insulin, HbA1c, serum lipids, and plasma cortisol were performed in a central laboratory. Insulin sensitivity was calculated as the homeostasis model assessment of insulin resistance index (HOMA-IR) by using the HOMA Calculator (Headington, Oxford, UK) (http://www.dtu.ox.ac.uk).

### 2.4. Sleep Quality

A total of 81 subjects with type 2 diabetes were divided into two groups according to the Pittsburgh Sleep Quality Index (PSQI), including a diabetes group with poor sleep quality and a diabetes group with good sleep quality [18]. PSQI is a self-reported questionnaire that assesses sleep quality and disorders. It contains seven component scores: subjective sleep quality, sleep latency, sleep duration, habitual sleep efficiency, sleep disturbances, use of sleeping medication, and daytime dysfunction. The sum of the scores for these seven components determined sleep quality classification: more than 7 points indicated poor sleep quality and less than or equal to 7 points indicated good sleep quality (the threshold of poor sleep quality was 7 points in Chinese people as reported [19]). All participants had 15 min to complete the questionnaire under the guidance of one trained researcher.

### 2.5. Continuous Glucose Monitoring

All 81 subjects with diabetes were evaluated using a CGMS for 72 hours (MiniMed system, Medtronic Inc., USA). Participants were obliged to input their capillary blood glucose four times a day for adjusting the CGMS. Three meals were required to be eaten between 7:00 and 7:30, 11:00 and 11:30, and 17:00 and 17:30, respectively. The sensor of CGMS was installed on day 0 and removed on day 3. The data provided from CGMS were obtained during day 1 and day 2 to avoid any interference due to installation and removal of the sensor. Furthermore, the glucose values recorded on day 1 and day 2 were averaged in order to avoid bias. The nocturnal nadir and prebreakfast glucose values were quantified by the averaged CGMS data from day 1 to day 2. The magnitude of dawn phenomenon (ΔDawn) was quantified by its increment from nocturnal nadir to prebreakfast glucose level, and the threshold of ΔDawn was determined to be 20 mg/dL (1.11 mmol/L) according to Monnier et al. [3]. If all glucose measurements at night were higher than the prebreakfast measurement, ΔDawn level was considered to be 0.

### 2.6. RNA Preparation and Real-Time Quantitative PCR

Blood samples were taken from the forearm vein at 09:00 [7]. Leucocytes were isolated immediately using lymphocyte separation media (Dakewe Biotech, Shenzhen, China). The isolation of total RNA was performed using the RNAiso Plus reagent (TaKaRa, Otsu, Japan) according to the manufacturer's instructions. 1 *μ*g of total RNA was treated with RNase-free DNase I for 10 minutes at 37°C to remove any contaminating DNA and reverse-transcribed with AMV reverse transcriptase (TaKaRa, Otsu, Japan). Gene expression was analyzed by real-time quantitative PCR using the ABI PRISM 7000 Sequence Detection System (Applied Biosystems). The GenBank accession numbers, assay ID, and the target exons were as follows: NM_001178.4 and Hs00154147_m1, 9-10 for *BMAL1*; NM_002616.1 and Hs00242988_m1, 22-23 for *PER1*; NM_022817.1 and Hs00256143_m1, 8-9 for *PER2*; and NM_016831.1 and Hs00213466_m1, 15-16 for *PER3*. Relative mRNA levels of each transcript were determined by the comparative Ct method with the reference gene (GAPDH).

### 2.7. Statistical Analysis

Continuous variables were expressed as the mean ± SD and were analyzed using ANOVA and post hoc analysis for normally distributed data. HbA1c measurements were reported in International Federation of Clinical Chemistry and Laboratory Medicine (IFCC) units (mmol/mol) followed by derived National Glycohemoglobin Standardization Program (NGSP) units (%). Categorical variables were expressed as numbers (%) and analyzed by using the chi-squared test and Fisher's exact test. Differences in clock gene expression were analyzed between the diabetes group with poor sleep quality, diabetes group with good sleep quality, and normal controls using the Mann–Whitney test. Correlations were calculated using Spearman's correlation coefficient. All *P* values were 2-tailed and *P* values less than 0.05 were considered significant. The enter method of multiple linear regression analysis was used to determine the association of ΔDawn level with age, BMI, PQSI, and expression levels of *PER1*, *PER2*, *PER3*, and *BMAL1*. Variables were entered into the model if the significance of the *F* value was less than 0.05 and were removed if it was more than 0.1. Statistical analyses were performed with SPSS version 13.0 (SPSS Inc., Chicago, IL, USA). All enrolled subjects were identified by participant numbers in the database to ensure anonymity. All artworks were created by GraphPad Prism 5 (GraphPad Software Inc., San Diego, CA).

## 3. Results and Discussion

### 3.1. Clinical Characteristics

There were, in total, 81 subjects with diabetes and 28 normal controls (15 males and 13 females) enrolled in the study. All participants had completed the whole study. The clinical characteristics of the 109 participants are summarized in Table 1. According to the Pittsburgh Sleep Quality Index, 81 subjects with diabetes had been separated into two groups: 37 with poor sleep quality (PSQI more than 7 points, 21 males and 16 females) and 44 with good sleep quality (PSQI less than or equal to 7 points, 24 males and 20 females). Gender, age, body mass index, blood pressure, fasting insulin, postbreakfast insulin, HOMA-IR, lipid profile, and plasma cortisol level showed no significant differences between the two groups. When compared with normal controls, the diabetes group with poor sleep quality showed a significantly higher level of systolic blood pressure, diastolic blood pressure, fasting glucose, HbA1c, and HOMA-IR (all *P* < 0.01).

### 3.2. Analysis of 24 h Glycemic Profile and Dawn Phenomenon

**Figure 1:**
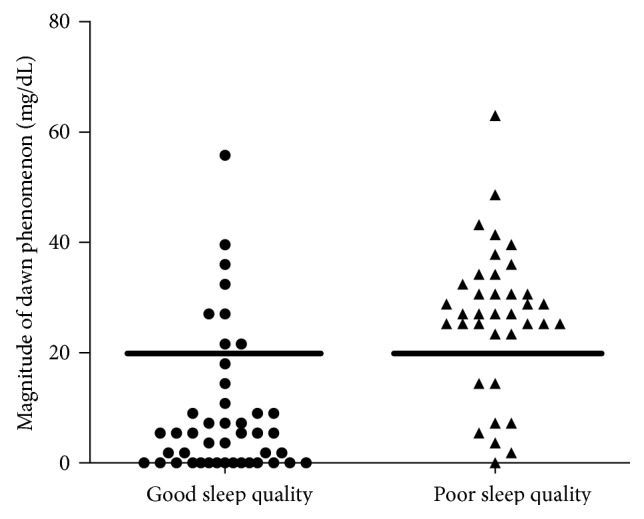
Magnitude of dawn phenomenon in subjects with diabetes. Black circle: diabetes group with good sleep quality; black triangle: diabetes group with poor sleep quality. When the threshold was settled at 20 mg/dL, the prevalence of dawn phenomenon in the two groups was 18.2% versus 78.4% (*P* < 0.001).

All subjects with diabetes were evaluated over 3 days using a CGMS. The 24 h glycemic profile, nocturnal nadir glucose, and magnitude of the dawn phenomenon (ΔDawn) are summarized in Table 1. The diabetes group with poor sleep quality presented with higher fasting glucose, postbreakfast glucose, 24 h mean glucose level, and HbA1c level than that with good sleep quality (all *P* < 0.05). The glucose levels at prelunch, postlunch, predinner, postdinner, and nocturnal nadir showed no statistical difference between the two diabetes groups. Following the determination of a ΔDawn threshold as 20 mg/dL, 37 of 81 subjects with diabetes were categorized as sufferers from dawn phenomenon; thus, the prevalence of dawn phenomenon in our study is 45.7%. The diabetes group with poor sleep quality had a higher ΔDawn level (*P* = 0.001) and higher proportion (*P* < 0.001) of dawn phenomenon than that with good sleep quality (see Figure 1 and Table 1).

### 3.3. Gene Expression Assays

**Figure 2:**
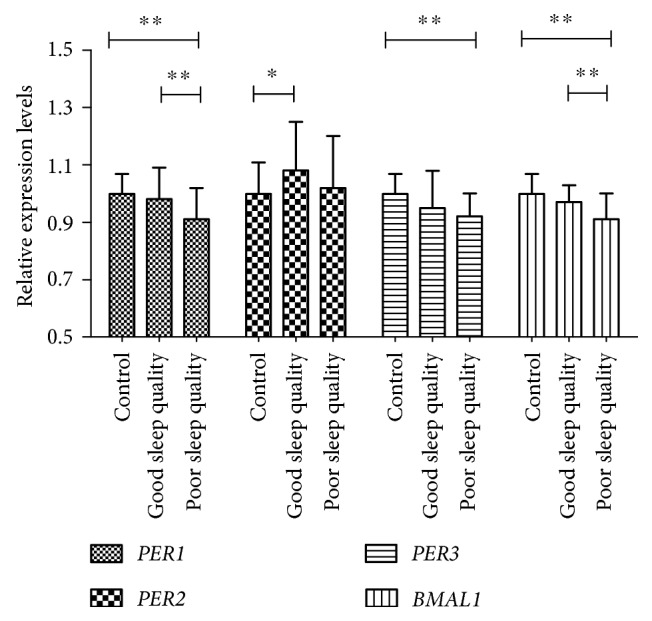
Transcript levels of the circadian clock genes in peripheral leucocytes of subjects with diabetes and normal controls. Peripheral leucocytes were obtained from 28 healthy controls, 37 diabetes subjects with poor sleep quality, and 44 diabetes subjects with good sleep quality at 09:00 hours. Transcript levels of the circadian clock genes were determined by real-time quantitative PCR. The mean value of the healthy control was set to 1 for each gene. ^∗^*P* < 0.05, ^∗∗^*P* < 0.01. The errors bars represented SD.

The transcript levels of these four clock genes (*BMAL1*, *PER1*, *PER2*, and *PER3*) in subjects with diabetes and normal controls were compared, as shown in Figure 2. The transcript levels of *BMAL1*, *PER1*, and *PER3* were significantly lower (all *P* < 0.01) in the diabetes group with poor sleep quality compared to the normal control group. Interestingly, the mRNA levels of *BMAL1* and *PER1* genes were found to be significantly lower (both *P* < 0.01) in the diabetes group with poor sleep quality compared to that with good sleep quality. In contrast, the transcript levels of the *PER2* and *PER3* had not display any significant difference between the two diabetes groups.

### 3.4. Correlation and Regression Analysis

**Figure 3:**
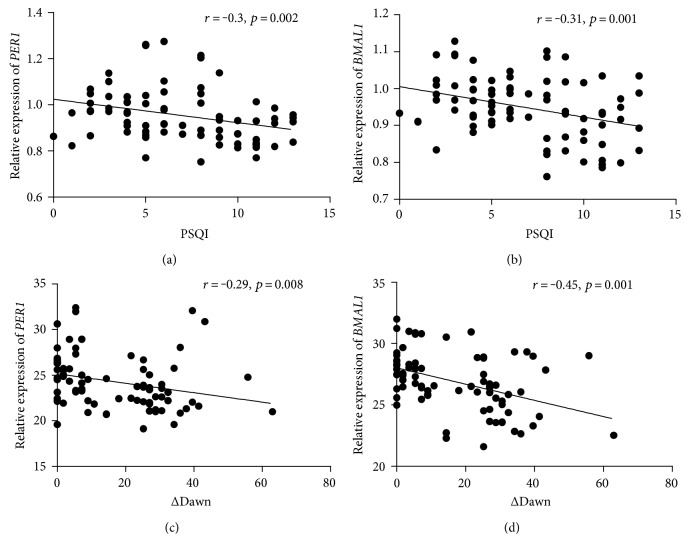
Relationships between Pittsburgh Sleep Quality Index and mRNA levels of *PER1* (a) and *BMAL1* (b). Relationships between ΔDawn and mRNA levels of *PER1* (c) and *BMAL1* (d).

The correlation analysis showed that PQSI is significantly correlated with ΔDawn level (*r* = 0.494, *P* < 0.001). Among the four circadian clock genes tested in our study, the expression of *PER1* and *BMAL1* was significantly negatively correlated with PQSI (*r* = −0.3, *P* = 0.002; *r* = −0.31, *P* = 0.001), as noted in Figures 3(a) and 3(b). Furthermore, the expression of *PER1* and *BMAL1* was significantly negatively correlated with ΔDawn level (*r* = −0.29, *P* = 0.008; *r* = −0.45, *P* = 0.001), as noted in Figures 3(c) and 3(d). Unfortunately, the level of HOMA-IR showed no correlation with either PQSI (*r* = 0.096, *P* = 0.319) or ΔDawn level (*r* = −0.095, *P* = 0.401).

The multiple linear regression analysis enter method was used to determine the association of ΔDawn with age, BMI, PQSI, and gene expression levels of *PER1*, *PER2*, *PER3*, and *BMAL1*. Multiple variable linear regression showed that PQSI and expression level of *PER1* were significantly independently correlated with ΔDawn level (adjusted *R*^2^ = 0.325, *P* = 0.001). The standard *β*-coefficients from the regression model of PQSI and *PER1* were 0.112 and −0.086, respectively.

## 4. Discussion

The term “dawn phenomenon” has been in use for more than 30 years, but endocrinologists seem to be uninterested in this area of study. With the development and wide use of CGMS, more and more scholars begin to pay attention to this phenomenon. Monnier et al. studied dawn phenomenon in three groups of subjects with type 2 diabetes (on diets alone, on insulin sensitizers alone, and on insulin secretagogues alone or in combination with insulin sensitizers) by using CGMS. The approximate impact of dawn phenomenon on HbA1c level was 0.4%, and the impact on the averaged 24 h mean glucose concentrations was 12.4 mg/dL [3]. Interestingly, their study showed a similar ΔDawn level in all three groups. These results revealed that dawn phenomenon was important in the management of type 2 diabetes and that it could not be eliminated by any of the current oral antidiabetes agents. This means that dawn phenomenon could only be controlled by using insulin, especially by using basal insulin such as neutral protamine Hagedorn (NPH), long-acting insulin analogs (LA-IA), or continuous subcutaneous insulin infusion (CSII) [20]. However, hypoglycemia occurred frequently in this population using CSII or using NPH as reported. This seems to be an inevitable side effect of using insulin in the treatment of dawn phenomenon [21, 22]. Thus, it is important for us to determine the mechanism leading to dawn phenomenon and to find a new way to manage fasting hyperglycemia.

In our study, 37 of 81 subjects with type 2 diabetes suffered from dawn phenomenon, and the prevalence of dawn phenomenon was 45.7%. After grouping the subjects by a PQSI of more than 7 points, the mean ΔDawn in the diabetes group with poor sleep quality was 26.5 ± 13.1 mg/dL, significantly higher than that in the diabetes group with good sleep quality (14.4 ± 12.8 mg/dL, *P* = 0.001). The diabetes group with poor sleep quality had a dramatically higher proportion of subjects with dawn phenomenon than that with good sleep quality (78.4% versus 18.2%, *P* < 0.001). Furthermore, correlation analysis indicated a significant relationship between sleep quality and ΔDawn level. Multiple variable linear regression also showed that sleep quality was significantly independently correlated with ΔDawn level after adjusting for age and BMI. It was very interesting to find that subjects with poor sleep quality presented with a higher fasting glucose, postbreakfast glucose, 24 h mean glucose level, and HbA1c levels than those with good sleep quality, while the glucose level at prelunch, postlunch, predinner, postdinner, and nocturnal nadir showed no significant difference between the two groups in our study. We could speculate that poor sleep quality has a greater influence on fasting and postbreakfast glucose than on other glucose levels.

It has been well published that sleep disturbances, sleep insufficiency, or sleep fragmentation has a relationship with abnormal glucose metabolism and increased diabetes risk. Prospective studies had revealed that sleep disorders were associated with an increased risk of incident diabetes [23]. However, few studies have presented a link between poor sleep quality and dawn phenomenon. Monnier et al. studied eighty-one individuals with type 2 diabetes and divided them into three subgroups by age (group 1: ≥70 years, group 2: 60–69 years, and group 3: ≤59 years). HbA1c levels showed no statistical significance across the three groups, while ΔDawn levels were different in the three groups: 22.0 ± 4.7 mg/dL in group 1, 21.3 ± 3.6 mg/dL in group 2, and 18.0 ± 3.6 mg/dL in group 3 [2]. The results argued that dawn phenomenon was present in the elderly but that this population might be frequently suffered from sleep disturbance. A study in China showed that dawn phenomenon was closely associated with obesity and insulin resistance, and the frequency of dawn phenomenon increased with body mass index [24]. It has been well known that obesity is correlated with OSAHS, which combines hypoxemia and sleep fragmentation and is a major risk factor for insulin resistance and possibly diabetes [23]. Furthermore, a human study showed that exogenous growth hormone injection in the early evening decreased the metabolic clearance rate of insulin and reduced insulin sensitivity in subjects with type 2 diabetes. The results provided direct evidence for the role of the growth hormone, which is secreted mostly during sleep, in regulation of insulin sensitivity and in causation of dawn phenomenon in subjects with type 2 diabetes [5]. Another study in China provided direct evidence of the association between poor sleep quality and dawn phenomenon. Ren et al. [19] demonstrated that the levels of HbA1c, fructosamine, increment of fasting glucose and nocturnal nadir glucose, 24 h mean glucose, fasting insulin, HOMA-IR, and area under curve of insulin were significantly higher in the diabetes subjects with poor sleep quality than in those with good sleep quality. In addition, poor sleep quality was positively correlated with the glucagon-to-insulin ratio. These results suggested that poor sleep quality could be one of the main causes of dawn phenomenon and that dysfunction of both islets *α*-cell and *β*-cell was involved in this pathophysiology.

**Figure 4:**
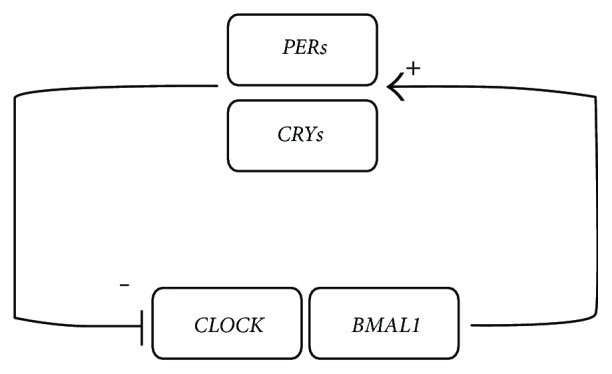
The core mechanism of a circadian clock gene. The *CLOCK*–*BMAL1* heterodimer is the core of circadian clock. It stimulates the transcription of period (*PERs*, including *PER1*, *PER2*, and *PER3*) and cryptochrome (*CRYs*, including *CRY1* and *CRY2*). Soon afterwards, *PERs* and *CRYs* translocate to the nucleus and inhibit *CLOCK*–*BMAL1* activity.

Circadian disruption has been considered to be associated with *β*-cell dysfunction, obesity, and type 2 diabetes in both animal studies and human studies [25]. Shift work, which has been regarded as a common cause of circadian disruption in modern society, has been proven to be correlated with an increased risk of type 2 diabetes by prospective cohort studies [26–28]. Circadian clock genes are important in the regulation of glucose homeostasis. The *CLOCK* and *BMAL1* complex is the transcription factor that acts as a positive regulator of circadian clock gene expression, such as regulation of the period and cryptochrome families, while period and cryptochrome proteins feedback and inhibit their own expression and other clock genes [29] (see Figure 4). Impairment of the circadian clock genes such as *CLOCK*, *BMAL1*, cryptochrome (*CRY1* and *CRY2*), and period (*PER1*, *PER2*, and *PER3*) contributes to the decrease in glucose tolerance, defective *β*-cell function, and the development of type 2 diabetes. Whole-body *BMAL1* knockout mice presented with diminished gluconeogenesis and could not recover from insulin-induced hypoglycemia [30]. Mice deficient of *PER2* had markedly increased circulatory levels of insulin and decreased insulin clearance compared with wild-type mice. Insulin secretion was more effectively stimulated by glucose in *PER2*-deficient mice [31]. Genetic loss of *CRY1*/*CRY2* results in decreased glucose intolerance, increased glucose level, and constitutively high levels of circulating corticosterone in mice [32]. In a human study, leucocytes sampled from subjects with type 2 diabetes expressed significantly lower transcript levels of *BMAL1*, *PER1*, and *PER3* compared with leucocytes from normal controls; moreover, transcript expression was inversely correlated with HbA1c levels [7].

In our study, the transcript levels of *BMAL1*, *PER1*, and *PER3* were also significantly lower in the diabetes groups compared to the normal control group. More importantly, the mRNA levels of *BMAL1* and *PER1* genes were found to be significantly lower in the diabetes group with poor sleep quality compared to that with good sleep quality. In addition, the expression of *PER1* and *BMAL1* was negatively correlated with ΔDawn level. To the best of our knowledge, this is the first study to discuss the relationship between dawn phenomenon and circadian clock genes. Poor sleep quality and the impairment of circadian clock seem to be closely involved in the pathophysiology of dawn phenomenon in type 2 diabetes. Because poor sleep quality is mostly a controllable ailment, it could be a new target for the management and treatment of dawn phenomenon and type 2 diabetes.

However, our study still has limitations that should be mentioned. The most important limitation is that it was a cross-sectional trial. It remains difficult to discern the “eggs and chickens” among the parameters that were tested. And we could not evaluate the long-term influence of poor sleep quality on dawn phenomenon. In addition, polysomnography has not been used in our study; thus, we could not exclude the influence of OSAHS on our results. Further interventional trials need to be designed. It would be interesting to demonstrate whether changes in sleep quality are or are not associated with changes in the magnitude of the dawn phenomenon and expression levels of circadian clock genes.

## 5. Conclusions

These results of our study showed that poor sleep quality is associated with dawn phenomenon in subjects with type 2 diabetes. Furthermore, mRNA expression of circadian clock genes such as *PER1* and *BMAL1* is dampened in peripheral leucocytes of diabetes subjects with poor sleep quality. Sleep quality and expression level of *PER1* are independent factors of dawn phenomenon. These results argued that cross talk between sleep quality, dawn phenomenon, and circadian clock gene may be an important pathophysiological mechanism in type 2 diabetes. Even though this study is observational in design, such results seem to indicate that poor sleep quality in type 2 diabetes could be responsible for both an overexpression of the dawn phenomenon and an underexpression of circadian clock genes.

## Figures and Tables

**Table 1:** Characteristics and glucose profiles of the study population, normal controls, and the diabetes group with poor sleep quality and with good sleep quality.

Variables	DM group with poor sleep quality	DM group with good sleep quality	^a^ *P* value	Control group	^a^ *P* value
No. (M/F)	37 (21/16)	44 (24/20)	0.565	28 (15/13)	0.421
Age (years)	66.2 ± 8.8	65.6 ± 10.3	0.774	64.43 ± 6.7	0.364
BMI (kg/m^2^)	24.8 ± 4.1	24.4 ± 3.0	0.588	23.2 ± 2.7	0.054
PSQI	10.l ± 1.7	4.1 ± 1.7	<0.001	4.0 ± 1.5	<0.001
Systolic BP (mmHg)	132.0 ± 13.1	129.9 ± 10.9	0.452	121.7 ± 13.7	0.003
Diastolic BP (mmHg)	78.7 ± 7.8	77.6 ± 7.0	0.498	73.0 ± 7.9	0.006
HbA1c (mmol/mol)	56 ± 5	53 ± 7	0.032	37 ± 4	<0.001
(%)	7.3 ± 0.5	7.0 ± 0.6		5.5 ± 0.4	
Fasting insulin (*μ*U/mL)	7.9 ± 4.5	8.3 ± 4.4	0.716	6.7 ± 3.0	0.189
Postbreakfast insulin (*μ*U/mL)	27.7 ± 16.1	25.9 ± 14.1	0.513	23.6 ± 14.2	0.325
HOMA-IR	2.6 ± 1.4	2.4 ± 1.5	0.674	1.5 ± 0.7	<0.001
Triglyceride (mmol/L)	1.8 ± 1.5	1.5 ± 1.3	0.357	1.5 ± 0.9	0.233
Total cholesterol (mmol/L)	4.6 ± 1.1	4.7 ± 1.2	0.682	5.0 ± 1.2	0.121
HDL-C (mmol/L)	1.3 ± 0.3	1.4 ± 0.3	0.301	1.3 ± 0.4	0.812
LDL-C (mmol/L)	2.4 ± 0.9	2.4 ± 0.9	0.933	2.8 ± 0.9	0.084
Plasma cortisol (nmol/L)	419.3 ± 89.5	384.1 ± 112.0	0.155	408.9 ± 85.3	0.673
Fasting glucose (mg/dL)	133.2 ± 21.6	120.6 ± 23.4	0.001	86.4 ± 10.8	<0.001
Postbreakfast (mg/dL)	217.2 ± 35.8	183.6 ± 22.1	0.032	N/A	N/A
Prelunch (mg/dL)	135.7 ± 32.6	127.9 ± 30.6	0.380	N/A	N/A
Postlunch (mg/dL)	162.3 ± 30.2	147.2 ± 28.44	0.056	N/A	N/A
Predinner (mg/dL)	134.3 ± 33.4	131.4 ± 33.5	0.814	N/A	N/A
Postdinner (mg/dL)	166.6 ± 28.2	155.7 ± 24.5	0.444	N/A	N/A
Nadir glucose (mg/dL)	106.5 ± 17.8	106.2 ± 18.2	0.642	N/A	N/A
24 h mean glucose (mg/dL)	175.2 ± 30.6	148.5 ± 20.7	0.028	N/A	N/A
ΔDawn (mg/dL)	26.5 ± 13.1	14.4 ± 12.8	0.001	N/A	N/A
Dawn phenomenon (%)	29 (78.4)	8 (18.2)	<0.001	N/A	N/A

Data are means ± SD or number (percentage).

^a^ *P* value compared to the DM group with poor sleep quality.

DM: diabetes mellitus; BMI: body mass index; BP: blood pressure; PSQI: Pittsburgh Sleep Quality Index; HDL-C: high-density lipoprotein cholesterol; LDL-C: low-density lipoprotein cholesterol; HOMA-IR: homeostasis model assessment of insulin resistance; N/A: not available; ΔDawn: difference between prebreakfast and nocturnal nadir glucose values; Dawn phenomenon: ΔDawn more than 20 mg/dL.
